# Supplementary material for: Identification and Validation of a Prognostic Signature for Thyroid Cancer Based on Ferroptosis-Related Genes
Source: Genes (Basel). 2022 Jun 1;13(6):997. doi: 10.3390/genes13060997 (PMC9222385; doi:10.3390/genes13060997)
Supplement: Supplementary file 1 [file genes-13-00997-s001.zip › genes-1697920-supplementary.pdf]

Table S1:The list for 259 ferroptosis-r3elated genes from the FerrDb database

SLC7A11	FH	UBC	SNORA16A	IREB2	NRAS	ZEB1
GPX4	CISD2	ALB	RGS4	HMGB1	KRAS	BLOC1S5-TXNDC5
AKR1C1	MIR9-1	TXNRD1	DPP4	ELAVL1	HRAS	CDKN2A
AKR1C2	MIR9-2	SRXN1	LOC390705	TFAP2C	LOC284561	PEBP1
AKR1C3	MIR9-3	GPX2	EIF2S1	SP1	SLC38A1	SOCS1
RB1	CBS	BNIP3	KIM-1	HBA1	SLC1A5	CDO1
HSPB1	ISCU	OXSR1	IL6	NNMT	GLS2	MYB
HSF1	ACSL3	CXCL2	SELENOS	PLIN4	GOT1	MAPK8
GCLC	OTUB1	RELA	ANGPTL7	HIC1	CARS1	MAPK9
NFE2L2	CD44	CHAC1	HSD17B11	STMN1	KEAP1	LINC00472
SQSTM1	BAP1	DDIT4	AGPAT3	RRM2	ATG5	PRKAA2
NQO1	BRD4	TFR2	SETD1B	CAPG	ATG7	PRKAA1
HMOX1	PRDX6	ASNS	TF	HNF4A	NCOA4	LINC00336
FTH1	MIR17	TSC22D3	FTL	NGB	ALOX12B	ABCC1
MUC1	SESN2	DDIT3	RPL8	YWHAE	ALOX15B	MIR6852
SLC3A2	NF2	JDP2	ATP5MC3	GABPB1	ALOXE3	ACVR1B
MT1G	ARNTL	SLC1A4	TFRC	AURKA	PHKG2	TGFBR1
SLC40A1	HIF1A	PCK2	MAFG	MIR4715	ACO1	EPAS1
CISD1	JUN	TXNIP	IL33	RIPK1	G6PDX	HILPDA
FANCD2	CA9	VLDLR	HAMP	PRDX1	ULK1	IFNG
FTMT	TMBIM4	GPT2	STEAP3	MIR30B	ATG3	ANO6
HSPA5	PLIN2	PSAT1	DRD5	CS	ATG4D	LPIN1
ATF4	MIR212	LURAP1L	DRD4	EMC2	BECN1	TNFAIP3
TP53	ATM	SLC7A5	MAP3K5	NOX1	MAP1LC3A	TLR4
HELLS	AIFM2	HERPUD1	MAPK14	CYBB	GABARAPL2	Fer1HCH
SCD	LAMP2	XBP1	SLC2A1	NOX3	GABARAPL1	YY1AP1
FADS2	ZFP36	ATF3	SLC2A3	NOX4	ATG16L1	EGLN2
SRC	PROM2	ZNF419	SLC2A6	NOX5	WIPI1	MIOX
STAT3	CHMP5	KLHL24	SLC2A8	DUOX1	WIPI2	TAZ
PML	CHMP6	TRIB3	SLC2A12	DUOX2	SNX4	MTDH
MTOR	CAV1	ZFP69B	GLUT13	G6PD	ATG13	IDH1
NFS1	GCH1	SIRT1	SLC2A14	PGD	ULK2	ATP6V1G2
TP63	PTGS2	VEGFA	EIF2AK4	PIK3CA	SAT1	FBXW7
CDKN1A	DUSP1	GDF15	ALOX5	FLT3	EGFR	PANX1
MIR137	NOS2	TUBE1	ALOX12	SCP2	MAPK3	DNAJB6
ENPP2	NCF2	ARRDC3	ALOX15	ACSL4	MAPK1	BACH1
VDAC2	MT3	CEBPG	ACSF2	LPCAT3	BID	LONP1

Table S2 List of overlaped genes

HELLS	ATG7	KLHL24	MUC1	NOX4
BNIP3	BECN1	CXCL2	MT1G	SLC38A1
RIPK1	EGFR	STEAP3	HSPA5	GLS2
**AKR1C3**	IDH1	PRKAA2	PML	ALOX15B
HMOX1	NFS1	SRC	CD44	MAP1LC3A
SLC40A1	TP63	HIF1A	ARNTL	SNX4
PLIN2	CDKN1A	PRDX1	JUN	**BID**
LAMP2	FH	LINC00472	VLDLR	ZEB1
PROM2	CISD2	**GPX4**	LURAP1L	DPP4
DDIT4	CBS	SCD	HERPUD1	CDKN2A
IREB2	BRD4	PGD	ZFP69B	MAPK8
HMGB1	SESN2	MAPK1	GDF15	TGFBR1
GABARAPL1	OXSR1	PEBP1	**MAP3K5**	ANO6
ISCU	TSC22D3	AKR1C1	ALOX5	MIOX
SLC1A5	XBP1	NFE2L2	ACSF2	**FBXW7**

Table S3. Prediction comparison of AKR1C3_clinical model and Clinical model.

	Survival	Non-survival	Total
Patients correctly predicted by two models	382	11	393
Patients that Clinical model can correctly predict but AKR1C3_clinical mode l cannot correctly predict	5	0	5
Patients that AKR1C3_clinical model can correctly predict but Clinical mode l cannot correctly predict	31	1	32
Patients who could not be correctly predicted by two models	64	4	68
Total	482	16	498

Table S4 Data cases involved in different logistic regression models

Model	Pathologic st age	T stage	N stage	Extrathyroidal extension	Residual tumor	AKR1C3	BID	FBXW7	GPX4	MAP3K5
1	√	√	√	√	√					
2						√	√	√	√	√
3	√	√	√	√	√	√				
4	√	√	√	√	√		√			
5	√	√	√	√	√			√		
6	√	√	√	√	√				√	
7	√	√	√	√	√					√
8	√	√	√	√	√	√	√	√	√	√

Table S5 Performance of different logistic regression models

Model	AUC	Accuracy	Precision	Recall	F1 score	Sensitivity	Specificity
1	0.775	0.799	0.987	0.803	0.886	0.803	0.688
2	0.772	0.647	**0.994**	0.639	0.778	0.639	**0.875**
3	0.816	**0.853**	0.990	**0.857**	**0.919**	**0.857**	0.750
4	0.805	0.705	0.991	0.701	0.821	0.701	0.812
5	0.792	0.831	0.988	0.836	0.906	0.836	0.688
6	0.828	0.813	0.990	0.815	0.894	0.815	0.750
7	0.790	0.803	0.987	0.807	0.888	0.807	0.688
8	**0.860**	0.807	0.992	0.807	0.890	0.807	0.812
